# Effect of pharmaceutical promotion and incentives offered by pharmaceutical companies on the prescribing pattern of medical students: a cross-sectional study from a developing nation Pakistan

**DOI:** 10.3389/fmed.2024.1334518

**Published:** 2024-05-23

**Authors:** Ali Hassan Gillani, Hafsa Arshad, Muhammad Farooq Umer, Muhammad Arshed, Farman Ullah Khan, Kamran Bashir, Sen Xu, Hasan Mujtaba, Mohamed Izham Mohamed Ibrahim, Yu Fang

**Affiliations:** ^1^Department of Pharmacy Administration and Clinical Pharmacy, School of Pharmacy, Xi’an Jiaotong University, Xi’an, Shaanxi, China; ^2^Center for Drug Safety and Policy Research, Xian Jiaotong University, Xi’an, Shaanxi, China; ^3^Shaanxi Centre for Health Reform and Development Research, Xi’an, China; ^4^Departement of Preventive Dentistry, College of Dentistry, King Faisal University, Hofuf, Alahsa, Saudi Arabia; ^5^University Institute of Public Health, Faculty of Allied Health Sciences, The University of Lahore, Punjab, Pakistan; ^6^College of Pharmacy, University of Sargodha, Sargodha, Pakistan; ^7^Department of Pathology Shaheed Zulfiqar Ali Bhutto Medical University, PIMS, Islamabad, Pakistan; ^8^Department of Clinical and Pharmacy Practice, College of Pharmacy, QU Health, Qatar University, Doha, Qatar

**Keywords:** pharmaceutical promotion, pharmaceutical companies, medical students, Punjab, Pakistan

## Abstract

**Background:**

Pharmaceutical companies continuously pursue healthcare professionals, starting from the medical college level, which can ultimately lead to irrational prescribing of drugs and antibiotics. Therefore, our main aim was to evaluate the opinions and attitudes of medical students toward pharmaceutical promotion.

**Methods:**

This study utilized a cross-sectional online survey that applied the snowball sampling technique. Data were collected from three public and three private sector medical colleges in Punjab, Pakistan using snowball sampling. A modified version of a pre-structured questionnaire was used to collect data between October 2020 and January 2021. Medical students from the third year onward were captivated. The tool was made available on Google Forms and students could access it by clicking the link shared. The effect of promotion on prescribing pattern and future prescribing of antibiotics were measured. Descriptive statistics, chi-square, and *t*-test were used to analyze the data.

**Results:**

A total of 1,301 students filled out the survey, but only 1,227 responses were acceptable. The average age was found to be 23.4 ± 1.59 years. Slightly more than half of the respondents were male participants (57.7%), and a significant proportion (84.1%) reported being aware of pharmaceutical promotion. A smaller number (27.7%) felt that physicians who meet medical representatives more frequently tend to prescribe more antibiotics and 46.3% indicated they would be willing to prescribe antibiotics under the promotional influence. Medical students who were male, in senior college years, attended government institutions, and had lower parental income showed significantly higher perception and attitude scores (*p* < 0.05) which, in turn, may show their inclination to promotional activities. Many students agreed with the view that pharmaceutical promotion (PP) activities may alter prescribing practices and also believed that they contribute to the increased irrational prescribing of drugs and antibiotics.

**Conclusion:**

The study revealed that only a small number of students are willing to engage in promotional activities and accept rewards, which influences their choice toward selection of drugs and antibiotics. This study highlighted the necessity of giving proper educational instructions regarding the promotion of drugs to medical students. This study also focused on the educational prerequisites of the students.

## Introduction

Pharmaceutical companies (PCs) continuously persuade doctors and pharmacists to prefer their medicines over others. PCs achieve their targets using a set of highly persuasive methods known as pharmaceutical promotions (PPs) (1). These tactics are informative and necessary for healthcare providers (HCPs) including pharmacists and doctors to update their knowledge about drugs. However, personalized advertising can influence the minds of prescribers and alter their prescribing behaviors, finally increasing drug costs (2, 3). It is highly evident that general practitioners who have a high tendency to accept gifts and who regularly meet with medical representatives (MR) are more inclined to recommend their medicines (4). Occasionally, data provided by PCs can be misleading and serve as a basis for improper prescribing (5). Therefore, every HCP should verify the information provided by MRs to ensure that it is unbiased and not solely aimed at making a profit while avoiding misleading decisions (6, 7). A meta-analysis of studies conducted in six countries found that PP influences physicians’ prescribing behaviors resulting in increased prescription costs and numbers (8). The spending on promotional strategies is substantial, surpassing research and development (R&D) expenses. The top 10 well-known global PCs increased their sales revenue by US $288 billion through personalized advertisements between 1996 and 2005, compared to a 21% income spending on R&D (9). In 2012 alone, approximately US $90 billion was spent on PP to establish relations between physicians and MRs to boost drug marketing. These promotional inducements are increasing significantly every day, with 80% of doctors receiving payments and other promotional incentives in 2018 totaling US $2.18 billion (10).

Medical students are the future decision-makers and hold an integral position in the community (11). They are continuously exposed to the influence of PCs, which aim to cultivate a positive attitude toward their products and their responsibilities (4). It is noteworthy that the initial interaction of the physician-in-training with MRs often occurs during the early years of medical college (12). In many instances, undergraduate medical students receive small gifts, such as calendars, pens, books, and free lunches, as a part of promotional offers (13). Medical students in Canada and the US receive many such gifts and their interaction with MRs is quite frequent; sometimes occurring up to 10 times per month (12, 14, 15). Critics argue that these intentional marketing strategies may influence the prescription-writing patterns of these students when they enter the professional world (16). The influence and behavior alteration are instilled at the school level with many students viewing receiving minor gifts from PCs as ethically acceptable. For example, 85% of undergraduate students deemed receiving a $50 gift inappropriate from politicians, but 46% considered the same amount appropriate when received from MRs (14). However, several students have identified a significant lack of training for undergraduates regarding their interactions with MRs (14). Substantial shortcomings have also been observed in students’ knowledge about drug marketing expenses, the accuracy of drug information, and professional ethics as presented by MRs (14, 17). These observations raise serious concerns about the potential influence of PP that may have on medical students. In the United States, several clinical and pre-medical students have reported involvement in advertising activities, and a survey of third-year students revealed that while they were aware of the negative effects of PP, most of them accepted gifts, and felt that it was appropriate to do so (18). This suggests that they are influenced by PP early in their educational career.

The lack of legislation and proper policy enactment in Pakistan contribute to the over-the-counter availability of antimicrobials, and promotion is playing a significant role in this phenomenon (1, 3). The pressure to comply with PP profoundly influences doctors’ prescribing practices and leads to increased antimicrobial drug usage (19). In our literature survey on PubMed, Medline, and Google and using keywords such as “medical students,” “Medical undergraduate,” “Education,” “attitudes,” “pharmaceutical promotion,” and “Pakistan” in various combinations to explore studies conducted in Pakistan, we did not find many influential studies in Pakistan on this topic except for one by Siddique UT and colleagues who reported that more than two-thirds of students were comfortable with receiving gifts from PCs (20). This finding highlighted a high tendency of gift acceptability and the pressing need to incorporate guidelines into the medical curriculum. The study was conducted in Karachi, Sindh, Pakistan, emphasizing the need for a similar study in Punjab, Pakistan. As part of the Pakistani medical education system, we have observed that MRs approach pharmacy and medical students during pharmaceutical exhibitions held on college premises or at conferences. These interactions are sometimes associated with the exchange of free meals and small gift items; however, the ethical impact of these transactions has not been investigated in Punjab previously. The only available study is almost a decade old. Therefore, to assess students’ exposure to PP, their attitudes toward and acceptance of industry marketing strategies and gifts, and the effect of gifts and incentives on their future prescribing patterns, we conducted a cross-sectional survey among medical students at six medical colleges in Punjab, Pakistan. We also evaluated whether these attitudes were influencing future prescribing practices for antibiotics.

## Materials and methods

### Study design and setting

Pakistan comprises four provinces (Punjab, Baluchistan, Sindh, and Khyber Pakhtunkhwa) and two independent administrative territories (Gilgit-Baltistan and Azad Jammu Kashmir). Provinces are further divided into divisions, districts, tehsils (administrative area consisting of towns), and towns. Punjab covers one-fourth (26%) of the total land area of Pakistan but is the most populous of all the provinces accounting for 60% of the population (21). There were approximately 44 medical colleges (private and government) operating in Punjab. This study was cross-sectional and was conducted in six medical colleges. These six medical colleges were selected based on ease of data collection (convenience sampling). All of the colleges had an average student batch size of 100 students in each class. A bachelor’s degree in medicine consists of a 5-year program with rigorous training in all aspects of medical knowledge followed by 1 year of residency training.

### Study participants and data collection

All female and male medical undergraduates currently enrolled in the third, fourth, or final year of study in medical colleges were targeted. We conducted this survey online because of partial and complete lockdowns during the COVID-19 outbreak. We opted for an online platform for data acquisition because it was challenging to conduct paper-based or observational surveys during the lockdown. In Pakistan, a large proportion (76 million people) frequently use the internet, with 37 million actively using various social media platforms (18). We employed snowball sampling, and the survey was made available on Google Forms from October 2020 to January 2021. Initially, we contacted students from selected colleges via phone with the assistance of their class directors. Subsequently, we shared the online link to the survey instrument with students and instructed them to share it with as many other students as possible. The online questionnaire began with a brief study introduction, information about privacy, a consent statement, the right to withdraw, and voluntary participation on the first page. Participants could then access the content of the study questionnaire by clicking the provided link. Participants were able to answer all of the questions by simply clicking on each question. To increase survey participation, a reminder was sent to the main students 2 weeks after the initiation of the survey. Survey answers were collected anonymously.

### Study tool development

An extensive literature review was conducted to identify related studies from Pakistan and around the world. To the best of our knowledge, we found only a few studies on the presented topic (4, 20, 22–27). Many studies were available regarding medical student’s interaction with PCs worldwide, but only one study was available in Pakistan. A comprehensive 55-item instrument was developed based on the literature mentioned above. Most of the questions were adopted from survey tools previously used for medical students (20, 22–27). The questionnaire sought information in four main sections: Section 1: demographics of medical students (9 items); Section 2: perception of medical undergrads about PP (10 Items); Section 3: attitudes/behaviors of medical students toward PP, policies, or guidelines regarding interactions with PCs and MRs, inadequacies in the medical curriculum, and the impact of PP on future drug and antibiotic prescribing by students (31 items); and Section 4: The final part contained one question about the acceptance of gifts, a scenario mentioning students’ willingness to accept a portion of the fee upon attending a conference, a statement about the role of MRs, and two open-ended questions gathering information on MRs interaction with students, and the role of these interactions in completing the questionnaire. As there were no legal rules and regulations in our medical setting regarding interactions between PCs and medical students, AMSA guidelines were followed. The detailed questionnaire is provided in the Supplementary file 1. Only the principal investigator had access to the Google account, and the data analysis file was directly imported from the site. After conceptualizing the tool, the validity of its content was tested by two professors with a background in pharmaceutical practice. Minor changes were made to our research questionnaire, which included converting US dollars ($) to Pakistani Rupees (PKR) in parental income and finally adding two open-ended questions. This modified version is then pre-tested before being offered to real participants. The pilot study was conducted, directing the tool to 10 students from each institution. During the pre-test period, students were asked to report back about understanding the questions, the order of the questions, an explanation, and the time they spent answering a full questionnaire. Items were assessed to be unambiguous and socially apt; no difficulties were observed, and hence, no additional changes were made. The mean average time for students to fill out the complete questionnaire was 15 min.

### Data analysis

All items in part 2 (Perception) and part 3 (Attitude) were calculated on several 5-point Likert scales (5 = strongly agree, 4 = agree, 3 = neutral, 2 = disagree, and 1 = strongly disagree). Cumulative scores were calculated for the perception and attitudes section to describe medical students’ perceptions and attitudes toward PCs and PPs and incentives in general. The new grading was unique and based on students’ answers to individual questions. Students who are more inclined to PP, and PCs get higher scores and vice versa. Some questions have negative consequences, so the answers strongly disagree give the highest score. Answers which show promising attitudes toward medical student–MRs interactions were given a score of 5 for strongly agree and 4 for agree, neutral responses were given a score of 3, and responses showing strict behavior toward drug companies were awarded 2 and 1 (disagree and strongly disagree, respectively). The reciprocal was used for negative items, i.e., strongly disagree =5 and strongly agree = 1. The collective score of each student was then measured. The data were imported from Google Forms as an Excel file. The data were then transferred to SPSS version 21.0. Accuracy and completeness were cross-checked by two investigators (AHG and HA).

A simple frequency test was used for demographics and individual questions. We used the chi-square test to evaluate the significant differences among the demographics and other variables with each individual perception and attitude statement. We also used independent *t*-tests and analysis of variance to compare scores between groups of medical students from different population groups, i.e., sex, grade, medical school, parents’ monthly income, parents belonging to a medical profession, and other independent variables (perception and attitude scores). The minimum score for the perceptual element is 10, and the maximum is 50. Similarly, for attitude items, the calculated minimum score is 31, and the maximum score is 155. A usual significance level of *p* < 0.05 was used to define the significance association in all the above tests.

### Ethical approval

Both the Bioethics Committee of Xi’an Jiaotong University (2021-19-PA) and the Ethics Committee of Superior University Lahore have given ethical approvals. Each student’s prior approval is also made in writing on the first page before starting the survey. All students must click the accept button before proceeding to the survey tool. None of the personal information (name, address phone number, etc.) was asked purposefully, and each participant was assured that the data would only be used for research purposes and would be kept confidential.

## Results

### Demographics information

The survey online link was dispersed among almost all the medical students of selected classes but only 1,301 completed it. In total, 74 responses were incomplete or were not filled by students. We received 1,227 acceptable and completely filled questionnaires. The average age was 23.4 ± 1.59 years, and more than half (57.7%) of the respondents were male participants. More than two-thirds of the respondents (68.7%) were students of government college, and almost the same number was from the final year (66.3%). A significantly higher proportion of the students (84.1%) heard of PP and 33.6% never heard of DTCA (Table 1).

**Table 1:** Demographic characteristics of 1,227 medical students.

No	Demographic information and statements	Number	Percentages
1	Sex		
	Male	708	57.7
	Female	519	42.3
2	Age (Mean ± SD) 23.4 ± 1.59		
3	Year of schooling		
	3rd	175	14.3
	4th	238	19.4
	Final year	814	66.3
4	Institution		
	Private medical college	384	31.3
	Government medical college	843	68.7
5	Approximate parental income PKR (monthly)		
	Less than 30,000	46	3.7
	30,000–50,000	407	33.2
	50,001–100,000	456	37.2
	More than 100,000	318	25.9
6	Do you have any parent(s) who is a medical doctor?		
	No	1,123	91.5
	Yes	104	8.5
7	Do you have at least one parent working for the pharmaceutical industry?		
	No	1,064	86.7
	Yes	163	13.3
8	Have you heard about pharmaceutical promotion for prescription drugs?		
	No	195	15.9
	Yes	1,032	84.1
9	Have you heard about direct-to-consumer advertising (DTCA) for prescription drugs?		
	No	412	33.6
	Yes	815	66.4

**Figure 1:**
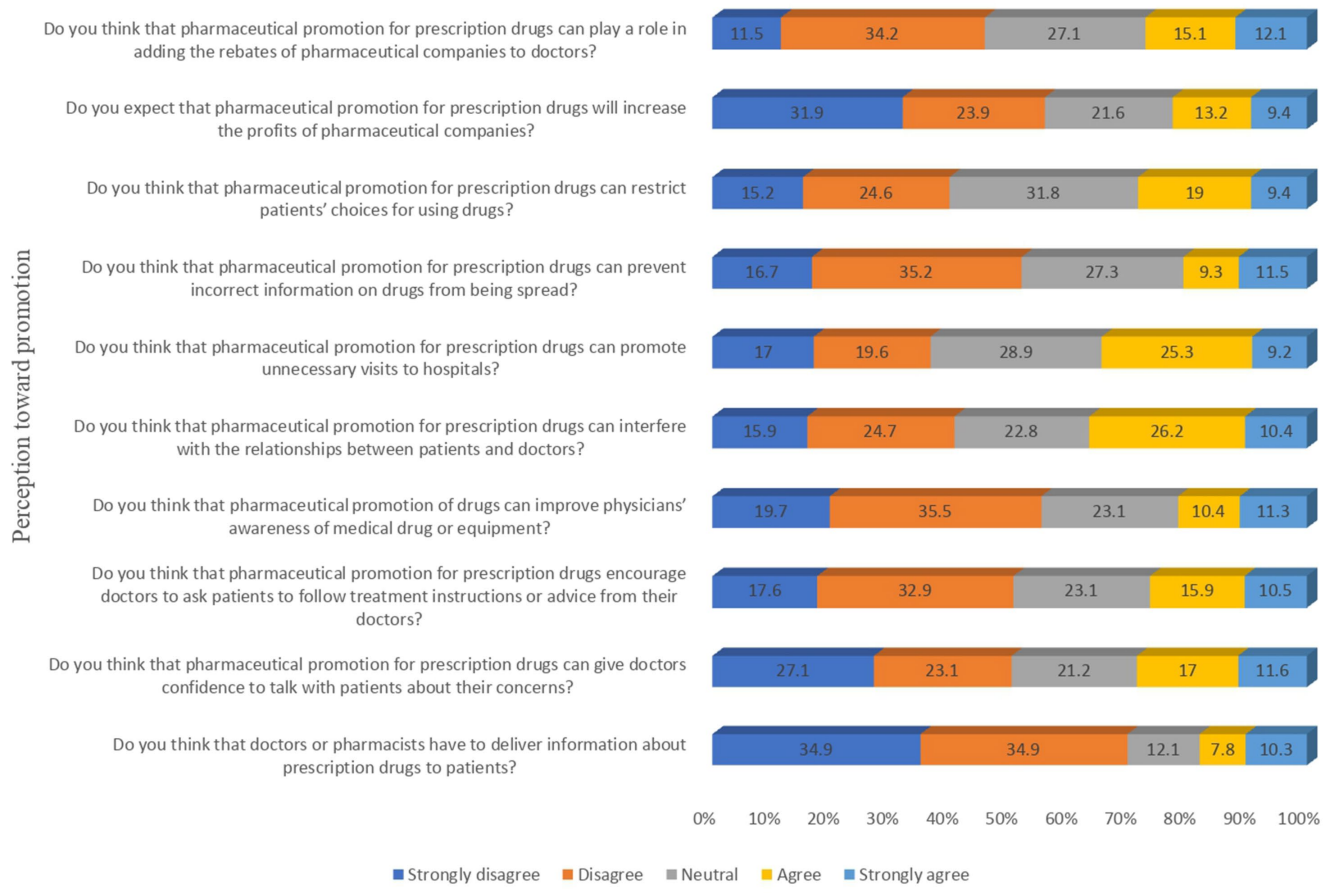
Responses of medical students towards perception items.

Figure 1 depicts the counts of the medical students toward perceptions regarding PP items. As per the results, a very small proportion of students (18.1%) expressed agreement (strongly agree and agree) that doctors or pharmacists should deliver the information in PP to patients and 28.6% said PPs give doctors the confidence to counsel patients. The more pressing concerns were that 28.4% of participants agreed that PP limits the doctor’s choice for medicines and 27.7% feel that PP is adding rebates for PCs to doctors. The responses to each Likert item were then summed up, and the mean score was 25.79 ± 7.29 with a minimum score of 13 and a maximum score of 46 (after inversing the score of negative items).

**Figure 2:**
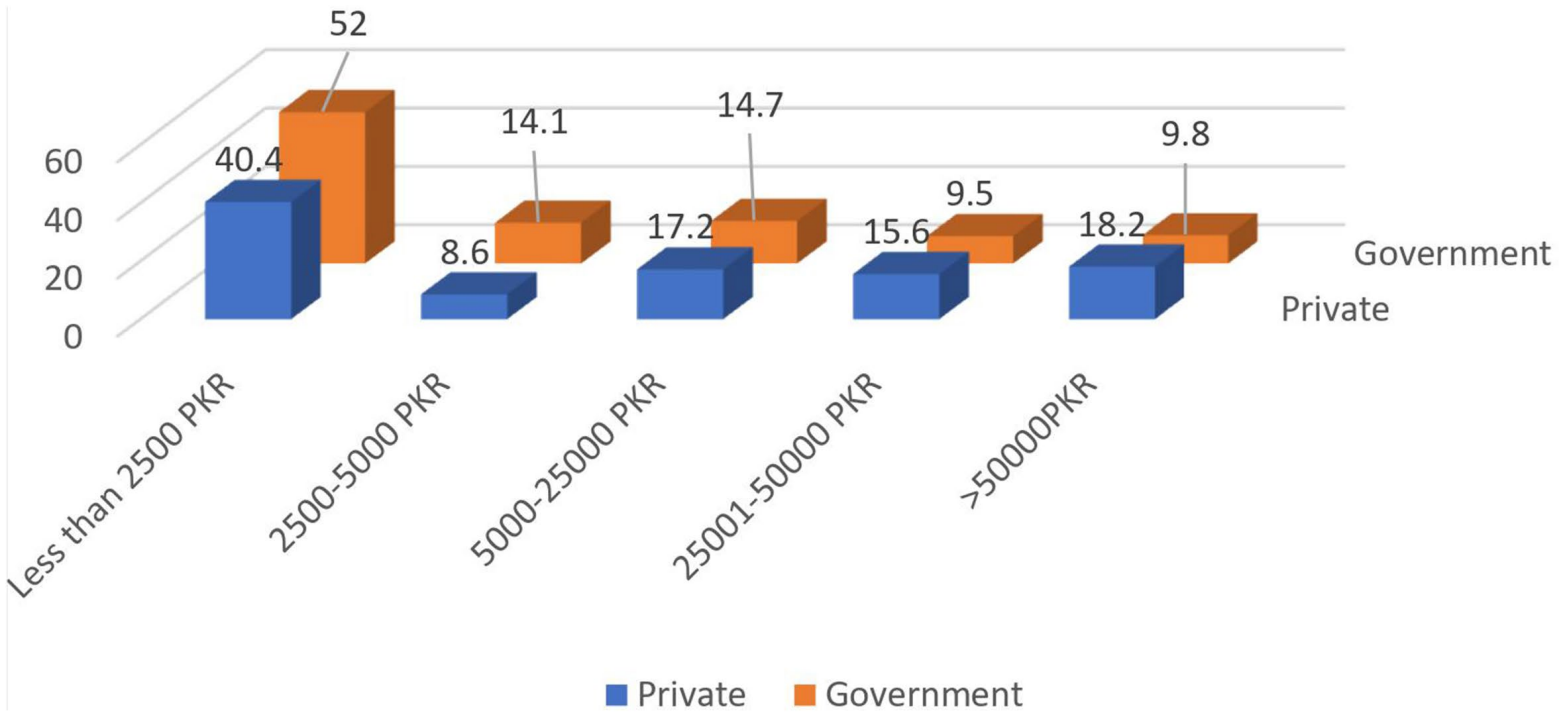
Acceptability of gifts with the monetary value among private and government institute students.

**Figure 3:**
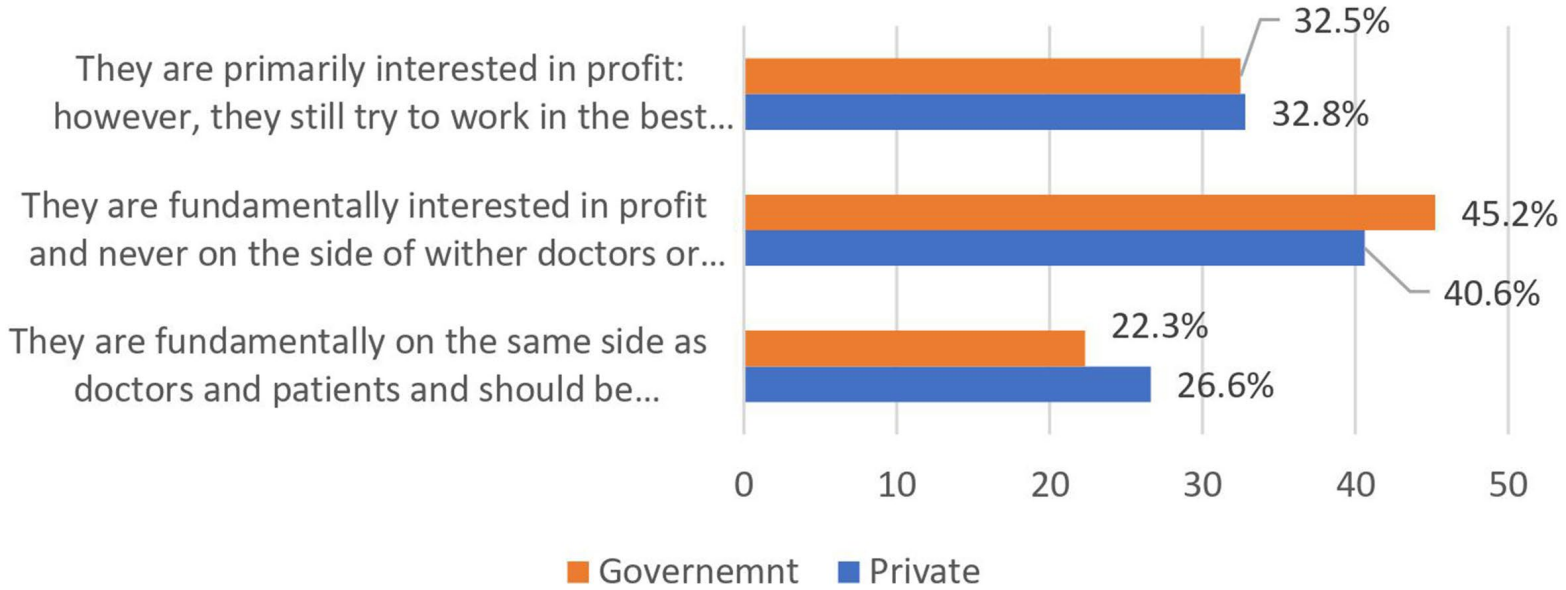
Difference between private and government medical students in response to statements they think are most true of drug companies.

The average for the attitude responses was 96.58 ± 8.45 with the range of score 79–123. Approximately 1 out of 3 (30.7%) students thought that PP is necessary for medical doctors, and 1 out of 5 (21.3%) students were willing to utilize data by PCs for the future counseling of patients. Slightly more than a quarter of students (27.1%) said PP should be regularized and 15.1% thought MRs should have a certificate to execute their profession. Few students (28.4%) complained that they were not taught enough to handle PP and MRs, and 44.6% disregarded that the syllabus provided them enough knowledge to interpret the knowledge given in PP (Figures 2, 3).

Related to the association of the PP with the present prescribing practices of doctors and their future prescribing, 46.3% of students said yes, they may prescribe antibiotics under the promotional influence in the future, and 45.9% of students said they may prescribe antibiotics under the gift pressure. Views of the students were less harsh in terms of other doctors’ prescribing behavior: 27.7% said doctors who meet representatives more often prescribe more antibiotics, and 26.2% said doctors who accept more gifts from companies prescribe more antibiotics than others (Table 2).

**Table 2:** Attitudes of 1,227 medical college students about pharmaceutical promotion.

Q no	Statement	1	2	3	4	5
1	Do you think that pharmaceutical promotion for prescription drugs is necessary for Physicians?	206 (16.8)	347 (28.3)	297 (24.2)	215 (17.5)	162 (13.2)
2	Do you think that information about drugs provided by pharmaceutical sales reps is reliable?	91 (7.4)	398 (32.4)	424 (34.6)	201 (16.4)	113 (9.2)
3	Are you willing to actively utilize the data obtained from pharmaceutical promotion for prescription drugs when consulting patients in the future?	69 (5.6)	531 (43.3)	365 (29.8)	183 (14.9)	79 (6.4)
4	Are you willing to actively accept patients’ opinions when they ask you to prescribe, fill, or administer drugs that they have knowledge of due to pharmaceutical promotion in the future?	135 (11.0)	368 (30.0)	354 (28.9)	277 (22.5)	93 (7.6)
5	Do you think that pharmaceutical promotion for prescription drugs should not be permitted on the websites of drug companies?	59 (4.8)	341 (27.8)	400 (32.6)	288 (23.5)	139 (11.3)
6	Do you think that pharmaceutical promotion for prescription drugs can create unrealistic expectations about drugs?	193 (15.7)	396 (32.3)	363 (29.6)	221 (18.0)	54 (4.4)
7	Do you think that pharmaceutical promotion for prescription drugs can play a part in improving patients’ drug compliance?	135 (11.0)	384 (31.3)	455 (37.1)	183 (14.9)	70 (5.7)
8	Do you expect that pharmaceutical promotion for prescription drugs will lead to increasing drug prices due to the cost of marketing strategies?	210 (17.1)	287 (23.4)	363 (29.6)	299 (24.4)	68 (5.5)
9	Do you think that the government should mandate preapproval of all pharmaceutical promotions for prescription drugs if they are permitted?	214 (17.4)	544 (44.3)	297 (24.3)	126 (10.3)	46 (3.7)
10	It is unacceptable for a physician to receive a gift from a drug company in any form	359 (29.3)	227 (18.4)	363 (29.6)	127 (10.4)	156 (12.3)
11	I would feel comfortable receiving the following gifts from a pharmaceutical company: lunch, penlight, stethoscope, textbook, watch/jewelry	162 (13.2)	238 (19.4)	321 (26.1)	207 (16.9)	299 (24.4)
12	Five drugs from five different companies are identical in terms of price, efficacy, and effectiveness. I would preferentially prescribe a drug from one of the companies that provided me with such gifts or incentives over those from companies that did not	138 (11.2)	194 (15.8)	261 (21.3)	291 (23.7)	343 (28.0)
13	Students should not have any interaction with drug companies in medical school	232 (18.9)	293 (23.9)	263 (21.4)	259 (21.1)	180 (14.7)
14	The information provided about drug effectiveness by pharmaceutical companies is untrustworthy	116 (9.5)	215 (17.5)	436 (35.5)	322 (26.3)	138 (11.2)
15	As long as their medications are accepted to be part of the standard care it is acceptable for physicians to be compensated PKR 100 by the drug company each time their drug is prescribed	94 (7.7)	214 (17.4)	490 (39.9)	164 (13.4)	265 (21.6)
16	It is acceptable for drug companies to sponsor events/educational seminars during medical school	264 (21.5)	366 (29.8)	299 (24.4)	169 (13.8)	129 (10.5)
17	If a drug company agreed to pay for the printing cost of all my class notes in undergraduate medical school, I would not mind the logo of that company appearing in the bottom corner of the first slide of my presentation lecture	139 (11.3)	229 (18.7)	355 (28.9)	296 (24.1)	208 (17.0)
18	Do you think these interactions should be regularized?	252 (20.5)	355 (28.9)	288 (23.5)	181 (14.8)	151 (12.3)
19	Do you think that the gifts and other things given by pharmaceutical industries to doctors should be recorded by the government as in many developed countries?	285 (23.3)	367 (29.9)	239 (19.5)	198 (16.1)	138 (11.2)
20	Do you feel that there is a need to incorporate guidelines regarding the relationship between the pharmaceutical industry and medical professionals in the undergraduate curriculum?	240 (19.6)	392 (31.9)	363 (29.6)	129 (10.5)	103 (8.4)
21	Do you think you have been taught enough about pharmaceutical promotion handling?	138 (11.2)	315 (25.7)	425 (34.6)	243 (19.8)	106 (8.6)
22	Do you feel that the syllabus provides you with enough knowledge about how to interpret the knowledge given during the promotional activity?	135 (11.0)	412 (33.6)	321 (26.2)	265 (21.6)	94 (7.6)
23	Do you think that pharmaceutical promotion for prescription drugs can have a negative effect on physicians’ prescribing practices?	105 (8.6)	314 (25.6)	369 (30.1)	349 (28.4)	90 (7.3)
24	Do you feel that these interactions with a representative are one of the key factors in the irrational prescribing of drugs?	161 (13.1)	398 (32.4)	335 (27.3)	208 (17.0)	125 (10.2)
25	Do you feel that these interactions with a representative are one of the key factors in the irrational prescribing of antibiotics?	228 (18.6)	272 (22.2)	393 (32.0)	244 (19.9)	90 (7.3)
26	You may prescribe antibiotics under the influence of the promotional activity in the future.	128 (10.5)	217 (17.7)	313 (25.5)	323 (26.3)	246 (20.0)
27	Do you feel that doctors who meet representatives more often prescribe more antibiotics?	208 (17.0)	308 (25.0)	372 (30.3)	224 (18.3)	115 (9.4)
28	Do you feel that those doctors who accept more gifts from companies prescribe more antibiotics than others?	299 (24.4)	301 (24.5)	306 (24.9)	174 (14.2)	147 (12.0)
29	You may prescribe antibiotics under the influence of acceptance of gifts by pharmaceutical companies.	139 (11.3)	248 (20.2)	277 (22.6)	267 (21.8)	296 (24.1)
30	Is there a need for a strengthening of ethical standards to control the interaction between physicians and pharmaceutical companies?	347 (28.3)	364 (29.7)	274 (22.3)	129 (10.5)	113 (9.2)
31	Do you think that MRs should have a certificate of professional and ethical capability to execute their profession?	382 (31.1)	305 (24.9)	355 (28.9)	80 (6.5)	105 (8.6)

5 = Strongly agree, 4 = Agree, 3 = Neutral, 2 = Disagree, 1 = Strongly disagree.

In case of demographic difference with individual perception items, male, students pursuing the fourth year of school, government institution, higher parental income (50,000–100,000PKR), at least one parent as a doctor, and at least one parent working for the company were showing significantly high perception score (*p* < 0.05). The same was the case reported with the attitude items except there was an insignificant relation observed in one of the parents as a doctor and few attitude items. (*p* < 0.05) (Supplementary Tables 1, 2).

The attitude and perception scores were analyzed by making various demographic subgroups of medical students. Perception scores of male participants, fourth-year students, students from government colleges, students whose parental income was <30,00PKR, and students who have at least one parent as a doctor were significantly higher than other students (*p* < 0.05). Among demographics, male sex, higher school year, students of government colleges, and those with low parental income, at least one parent as a doctor, and at least one parent working for PC depicted significantly more attitude scores (affinity toward attitude) (Supplementary Table 3).

More than half (52%) of the students from government colleges and 40.4% from private colleges felt comfortable accepting a gift worth <50,00PKR from the PC. In this study, nearly the same number of students from private and government colleges think that MRs were primarily interested in profit.

## Discussion

The objective of drug marketing initiates from the school years, with a deliberate focus on targeting students due to their adaptable and impressionable brains. The students’ perspectives on the PP indicated a clear requirement for further guidance and teaching. Historically, the majority of studies were conducted in high-income nations (2, 12, 16–18, 22–28), with only a few outliers in poor nations (4, 6, 15, 20). However, Pakistan lacks sufficient fundamental evidence about medical students’ engagement with MRs and the impact of PP on their future prescribing behavior. There is a lack of connection between the PP and the prescribing pattern of antibiotics and the impact of the PP on the irrational prescribing of antibiotics. Our earlier investigation demonstrated that pharmacists are distributing antibiotics in response to promotional pressure (3). Only one study conducted about a decade ago (20) in Karachi, Pakistan, by Siddique et al. has been found. In order to provide a comprehensive and lucid understanding of the impact of PP, we performed a survey in Punjab, Pakistan. The results of our study revealed a lack of comprehension among the students of the system, and only 25% of the students expressed the belief that physicians should not accept gifts from pharmaceutical corporations in any manner. This situation is concerning because it may lead to a future when physicians are more likely to engage in gift-taking conduct.

In this study, a relatively small percentage (30.7%) expressed the view that information in PP is essential for physicians, which is significantly lower compared to a study conducted in the US where half of the population agreed on the necessity of this information (27). Additionally, two-fifths of the medical students in our study expressed doubts about the reliability of this information, which is consistent with previous findings in Pakistan but lower than the percentages reported in the United States (70–90%). It is worth noting that the level of skepticism among government students in our study was not significantly high, whereas it was significantly high among private students in the Pakistani study (4, 14, 20, 28, 29). Approximately 50% of the public expressed reluctance to utilize this information in future patient counseling sessions. However, a significant majority of the Japanese population (73.3%) indicated their intention to incorporate the information provided by MRs into their future practices (28). When questioned about the expense, only 29.9% of our population concurred that this would result in an escalation in the cost of drug prescriptions, a finding that closely aligns with a survey conducted in the United States (27). In Italy, a significant number of students were cognizant of the financial impact of drug advertising, as evidenced by a majority of them being aware of its cost (30). Regarding the acceptance of gifts, 22.7% of physicians consider it unacceptable to take gifts in any form, whereas 41.3% expressed comfortable receiving items such as lunch, penlight, stethoscope, textbook, and watch/jewelry from PCs. Upon retrospective analysis, it is evident that the findings from the study conducted by Siddiqui et al. (20) align precisely with the results observed. However, a separate study conducted in Japan demonstrated a significantly higher level of receptiveness toward taking similar items as gifts (28). This discrepancy is justified by the fact that the majority of students cited financial difficulties or simply stated that others accept gifts (30, 31). Regarding the general acceptance of presents, there is a divergence of views between individuals and other decision-makers. In a poll, 85% of respondents expressed the belief that it is wrong for government officials to accept the same type of gift. This variation arises from the belief that they are immune to the influence of presents, whereas other professions alter their behavior in response to gifts (26).

When discussing ethical considerations, 27.3% of respondents believed that the government should keep a record of all gifts given to physicians, while others expressed the view that these contacts should be regulated. There is a division in the student’s behavior about physician–PC interaction being regularized by schools or the government. Kuwaiti and Italian studies ruled in favor of regularization, whereas the US study holds a contrasting view (4, 30, 32). Similarly, few respondents felt the need for guideline incorporation in the medical curriculum and have been taught enough about PP handling, and the syllabus provides enough knowledge to interpret the information given during the PP. This was in contrast to the previously conducted study in Pakistan, where a majority thought there was a need for guidelines incorporation (20), and the same is observed in Italy and the US (30, 32). This behavior is coupled with the increased demand for such education (12, 30). The difference in different areas of Pakistan is due to the difference in extra syllabi activities among the different medical universities. For example, the data from Karachi Agha Khan University showed much more compliance with international ethical practices than practices in Punjab hospitals.

Another topic we discussed was the impact of PP on the unreasonable prescription of medicines and antibiotics. Just over a third (35.7%) believed that PP has a detrimental impact on doctors’ prescribing practices, while a tiny percentage considered it to play a significant role in the inappropriate prescription of medications and antibiotics. The response rate was also low in the study conducted in Pakistan and the study conducted in Japan (20, 28). Nearly half of the respondents indicated that they would increase their prescription of medications and antibiotics when under the effect of the PP. No study has been undertaken to assess the impact of PP on medication and antibiotic prescribing. Our study found that 76.2% of participants believed that these promotions could lead to an increase in the irrational dispensing of antibiotics, and 18.6% admitted to dispensing antibiotics specifically because of these promotions (3). This suggests that there is a chain reaction starting from medical or pharmacy schools, and it is crucial to address this issue at its foundation. Our country lacks fundamental ethical guidelines that govern the practice of public policy and the connection between HCP and the PC. Furthermore, there is a deficiency in the proficiency required for engaging with MRs, and it has not been incorporated into the curriculum of medical schools. An appropriately structured curriculum is essential for preparing future doctors to effectively contribute to society (3). A strong correlation was found between educational years and parental income below 300,00PKR in relation to the cumulative perception and attitude scores. The most likely reason is that the disparity in the socio-economic backgrounds of the students may explain why they find it advantageous to take advantage of the free options offered by PC (20).

## Strength and limitations

This study, conducted among medical students in Punjab, Pakistan, is the first of its kind to explore the topic. It establishes a solid foundation by demonstrating the strong general preference of medical students for PP. Nevertheless, the deliberate and non-random selection of medical students for the study introduces a significant risk of selection bias. Students who possess a more favorable disposition toward promotion or ethical conduct may decline to participate. If such an event were to occur, there would be a greater likelihood and level of exposure to PP than what was observed in our study. Recall bias and reliance on self-reporting can also influence responses, instead of directly evaluating the actual activity. Furthermore, as this is a cross-sectional study, we cannot ascertain whether specific characteristics were causally linked to the long-term development of certain behaviors in medical students. Undoubtedly, intervention or prospective studies are necessary to elucidate such matters. A further limitation could be the lack of generalizability of the data, as the study was conducted exclusively in Punjab and only included six medical schools. Comprehensive nationwide surveys including many centers are necessary to have a thorough understanding of medical students.

## Conclusion

A minority of students believe that it is acceptable for physicians to receive presents, but a majority believe that it is acceptable for them to take gifts through a PC. Only a small number of students are willing to engage PP and they would be more inclined to prescribe additional antibiotics as a result of this influence. Although the aforementioned techniques are unconventional, a significant number of people do not get suitable instructions in the syllabus. This study focused on the educational prerequisites of the students. It is necessary to assign educators to provide training to students on the ethical aspects of relationships between HCPs and PCs as part of the formal medical curriculum. It is advisable to organize special seminars or lectures for students to acquire the tactics necessary to address the PP. The government of Pakistan ought to formulate and implement a comprehensive national strategy for the inclusion of MRs in medical colleges and pharmacy universities.

## Data availability statement

The raw data supporting the conclusions of this article will be made available by the authors, without undue reservation.

## Ethics statement

Bioethics Committee of Xi'an Jiaotong University (2021-19-PA) and the Ethics Committee of Superior University Lahore given ethical approvals. The studies were conducted in accordance with the local legislation and institutional requirements. The participants provided their written informed consent to participate in this study.

## Author contributions

AG: Conceptualization, Data curation, Formal analysis, Writing – original draft. HA: Conceptualization, Data curation, Formal analysis, Writing – original draft. MU: Data curation, Methodology, Writing – review & editing. MA: Conceptualization, Data curation, Methodology, Software, Writing – original draft. FK: Data curation, Software, Validation, Writing – review & editing. KB: Data curation, Investigation, Writing – review & editing. SX: Conceptualization, Formal analysis, Project administration, Writing – review & editing. HM: Data curation, Visualization, Writing – review & editing. MM: Software, Supervision, Writing – review & editing. YF: Funding acquisition, Project administration, Resources, Supervision, Writing – review & editing.
