# Supplementary material for: Effect of pharmaceutical promotion and incentives offered by pharmaceutical companies on the prescribing pattern of medical students: a cross-sectional study from a developing nation Pakistan
Source: Front Med (Lausanne). 2024 May 23;11:1334518. doi: 10.3389/fmed.2024.1334518 (PMC11153857; doi:10.3389/fmed.2024.1334518)
Supplement: Supplementary file 1 [file Table_1.docx]

**Table 1: Demographic association of 1227 medical students with individual perception item.**

| **No** | **Demographics** | **Q1** | **Q2** | **Q3** | **Q4** | **Q5** | **Q6** | **Q7** | **Q8** | **Q9** | **Q10** |
| --- | --- | --- | --- | --- | --- | --- | --- | --- | --- | --- | --- |
| **1** | **Gender** | 0.000 | 0.000 | 0.000 | 0.004 | 0.000 | 0.000 | 0.000 | 0.000 | 0.000 | 0.000 |
| 2 | **Year of study in the Pharmacy school** | 0.000 | 0.000 | 0.000 | 0.000 | 0.000 | 0.000 | 0.000 | 0.000 | 0.000 | 0.000 |
| 3 | **Institution** | 0.000 | 0.000 | 0.000 | 0.000 | 0.000 | 0.000 | 0.021 | 0.000 | 0.775 | 0.000 |
| 4 | **Approximate parental income** | 0.000 | 0.000 | 0.000 | 0.000 | 0.000 | 0.000 | 0.000 | 0.000 | 0.000 | 0.000 |
| **5** | **Do you have any parent(s) who is a medical doctor?** | 0.000 | 0.000 | 0.000 | 0.000 | 0.000 | 0.000 | 0.000 | 0.000 | 0.000 | 0.000 |
| **6** | **Do you have at least one parent working for the pharmaceutical industry?** | 0.000 | 0.000 | 0.000 | 0.000 | 0.000 | 0.000 | 0.000 | 0.000 | 0.066 | 0.000 |
| **7** | **Have you heard about pharmaceutical promotion for drugs?** | 0.000 | 0.011 | 0.000 | 0.000 | 0.000 | 0.000 | 0.000 | 0.000 | 0.000 | 0.000 |
| **8** | **Have you heard about direct-to-consumer advertising (DTCA) for prescription drugs?** | 0.000 | 0.000 | 0.022 | 0.000 | 0.000 | 0.000 | 0.000 | 0.011 | 0.000 | 0.000 |

**Chi square test = P value <0.05 considered significant**

**Table 2: Demographic association of 1227 medical students with individual attitude items.**

| **No** | **Demographics** | **Q1** | **Q2** | **Q3** | **Q4** | **Q5** | **Q6** | **Q7** | **Q8** | **Q9** | **Q10** | **Q11** | **Q12** | **Q13** | **Q14** | **Q15** | **Q16** | **Q17** | **Q18** |
| --- | --- | --- | --- | --- | --- | --- | --- | --- | --- | --- | --- | --- | --- | --- | --- | --- | --- | --- | --- |
| **1** | **Gender** | 0.006 | 0.000 | 0.000 | 0.000 | 0.000 | 0.000 | 0.000 | 0.000 | 0.188 | 0.000 | 0.000 | 0.000 | 0.000 | 0.000 | 0.000 | 0.000 | 0.000 | 0.000 |
| 2 | **Year of study in the Pharmacy school** | 0.000 | 0.000 | 0.000 | 0.000 | 0.000 | 0.000 | 0.000 | 0.000 | 0.000 | 0.000 | 0.000 | 0.000 | 0.000 | 0.000 | 0.000 | 0.000 | 0.000 | 0.000 |
| 3 | **Institution** | 0.031 | 0.244 | 0.00 | 0.052 | 0.000 | 0.000 | 0.000 | 0.000 | 0.000 | 0.152 | 0.000 | 0.021 | 0.000 | 0.000 | 0.000 | 0.000 | 0.000 | 0.000 |
| 4 | **Approximate parental income** | 0.000 | 0.000 | 0.000 | 0.000 | 0.000 | 0.000 | 0.000 | 0.000 | 0.000 | 0.000 | 0.000 | 0.000 | 0.000 | 0.000 | 0.000 | 0.000 | 0.000 | 0.000 |
| **5** | **Do you have any parent(s) who is a medical doctor?** | 0.000 | 0.004 | 0.000 | 0.004 | 0.000 | 0.011 | 0.002 | 0.000 | 0.000 | 0.000 | 0.000 | 0.000 | 0.321 | 0.55 | 0.007 | 0.015 | 0.000 | 0.000 |
| **6** | **Do you have at least one parent working for the pharmaceutical industry?** | 0.000 | 0.000 | 0.000 | 0.000 | 0.000 | 0.000 | 0.000 | 0.000 | 0.000 | 0.000 | 0.000 | 0.000 | 0.000 | 0.000 | 0.000 | 0.000 | 0.000 | 0.019 |
| **7** | **Have you heard about pharmaceutical promotion for drugs?** | 0.000 | 0.000 | 0.000 | 0.000 | 0.661 | 0.000 | 0.000 | 0.000 | 0.000 | 0.000 | 0.000 | 0.000 | 0.000 | 0.000 | 0.000 | 0.000 | 0.047 | 0.000 |
| **8** | **Have you heard about direct-to-consumer advertising (DTCA) for prescription drugs?** | 0.000 | 0.000 | 0.111 | 0.000 | 0.000 | 0.000 | 0.000 | 0.000 | 0.000 | 0.000 | 0.000 | 0.000 | 0.000 | 0.000 | 0.000 | 0.065 | 0.000 | 0.000 |
| **No** | **Demographics** | **Q19** | **Q20** | **Q21** | **Q22** | **Q23** | **Q24** | **Q25** | **Q26** | **Q27** | **Q28** | **Q29** | **Q30** | **Q31** |  |  |  |  |  |
| **1** | **Gender** | 0.000 | 0.000 | 0.000 | 0.000 | 0.000 | 0.000 | 0.000 | 0.000 | 0.000 | 0.045 | 0.000 | 0.000 | 0.000 |  |  |  |  |  |
| 2 | **Year of study in the Pharmacy school** | 0.000 | 0.000 | 0.000 | 0.000 | 0.000 | 0.000 | 0.000 | 0.000 | 0.000 | 0.000 | 0.000 | 0.000 | 0.000 |  |  |  |  |  |
| 3 | **Institution** | 0.000 | 0.000 | 0.000 | 0.000 | 0.000 | 0.183 | 0.000 | 0.000 | 0.000 | 0.000 | 0.000 | 0.000 | 0.000 |  |  |  |  |  |
| 4 | **Approximate parental income** | 0.000 | 0.000 | 0.000 | 0.000 | 0.000 | 0.000 | 0.000 | 0.000 | 0.000 | 0.000 | 0.000 | 0.000 | 0.000 |  |  |  |  |  |
| **5** | **Do you have any parent(s) who is a medical doctor?** | 0.000 | 0.062 | 0.000 | 0.000 | 0.000 | 0.000 | 0.000 | 0.000 | 0.000 | 0.000 | 0.000 | 0.000 | 0.000 |  |  |  |  |  |
| **6** | **Do you have at least one parent working for the pharmaceutical industry?** | 0.000 | 0.000 | 0.000 | 0.000 | 0.000 | 0.000 | 0.000 | 0.000 | 0.000 | 0.21 | 0.000 | 0.000 | 0.000 |  |  |  |  |  |
| **7** | **Have you heard about pharmaceutical promotion for drugs?** | 0.000 | 0.000 | 0.000 | 0.000 | 0.000 | 0.000 | 0.000 | 0.000 | 0.000 | 0.000 | 0.000 | 0.000 | 0.000 |  |  |  |  |  |
| **8** | **Have you heard about direct-to-consumer advertising (DTCA) for prescription drugs?** | 0.000 | 0.000 | 0.000 | 0.000 | 0.000 | 0.000 | 0.000 | 0.000 | 0.000 | 0.000 | 0.000 | 0.167 | 0.000 |  |  |  |  |  |

**Chi square test = P value <0.05 considered significant**

**Table 3: Association of perception score (10 items) and attitude score (31 items) with demographics values.**

| **No** | **Demographic** | **Perception score (10)** | | **Attitude score (31)** | |
| --- | --- | --- | --- | --- | --- |
|  |  | **Mean (SD)** | **P** | **Mean ± SD** | **P** |
| **1** | **Gender** |  |  |  |  |
|  | Male | 28.46 (7.51) |  | 97.04 (8.65) |  |
|  | Female | 26.40 (6.81) | 0.000 | 95.95 (8.12) | 0.027 |
| **2** | **Year of study in the Pharmacy school** |  |  |  |  |
|  | 3^rd^ Year | 27.69 (9.12) |  | 93.32 (4.01) |  |
|  | 4^th^ Year | 28.83 (5.32) |  | 96.84 (6.48) |  |
|  | Final Year | 27.20 (7.32) | 0.010 | 97.20 (9.45) | 0.000 |
| **3** | **Institution** |  |  |  |  |
|  | Private Medical College | 24.38 (7.34) |  | 95.78 (9.18) |  |
|  | Gov Medical College | 28.13 (7.21) | 0.000 | 96.94 (8.07) | 0.026 |
| **4** | **Approximate parental income** |  |  |  |  |
|  | < PKR 30, 000 | 40.0 (3.65) |  | 92.52 (3.26) |  |
|  | PKR 30,000 – PKR 50,000 | 28.52 (7.17) |  | 98.18 (6.97) |  |
|  | PKR50,001 – PKR 100,000 | 25.96 (6.55) |  | 96.47 (9.30) |  |
|  | > PKR 100,000 | 26.93 (6.90) | 0.000 | 95.27 (8.91) | 0.000 |
| **5** | **Do you have any parent(s) who is a medical Doctor?** |  |  |  |  |
|  | Yes | 32.81 (8.88) |  | 94.04 (8.67) |  |
|  | No | 27.10 (6.94) | 0.000 | 96.81 (8.39) | 0.001 |
| **6** | **Do you have at least one parent working for the pharmaceutical industry?** |  |  |  |  |
|  | Yes | 28.58 (8.92) |  | 93.48 (5.05) |  |
|  | No | 27.43 (7.00) | 0.061 | 97.05 (8.76) | 0.000 |
| **7** | **Have you heard about pharmaceutical promotion for drugs?** |  |  |  |  |
|  | Yes | 27.42 (6.90) |  | 97.04 (8.96) |  |
|  | No | 28.45 (8.79) | 0.071 | 94.13 (4.12) | 0.000 |
| **8** | **Have you heard about direct-to-consumer advertising (DTCA) for prescription drugs?** |  |  |  |  |
|  | Yes | 27.37 (6.98) |  | 96.53 (8.48) |  |
|  | No | 28.0 (7.87) | 0.152 | 96.66 (8.44) | 0.804 |

_Note: Q7 for perception items were negative so strongly disagree given 5 and strongly agree given 1 score_

_Note Q 5 6 9 10 13 14 18 19, 20, 22-25, 30,31 for attitude items were negative so strongly disagree given 5 and strongly agree given 1 score_
